# Public controversy and citizens’ attitude formation about animal research: A case for scholarship and recommendations on conflicts at the science-society interface

**DOI:** 10.1371/journal.pone.0295503

**Published:** 2024-01-03

**Authors:** Elena Link, Katharina Emde-Lachmund, Sophie Bruns, Anja Dittrich, Meike Stiesch, Axel Haverich, Stefan Treue, Christoph Klimmt

**Affiliations:** 1 Department of Communication, Johannes Gutenberg University, Mainz, Mainz, Germany; 2 Department of Journalism and Communication Research, Hanover University of Music, Drama, and Media, Hanover, Germany; 3 Dept. of Prosthetic Dentistry and Biomedical Materials Science, Hannover Medical School, Hanover, Germany; 4 Dept. of Cardiothoracic Surgery, Transplantation, and Vascular Surgery, Hannover Medical School, Hanover, Germany; 5 German Primate Center—Leibniz Institute for Primate Research, Goettingen, Germany; 6 Initiative Tierversuche Verstehen, Muenster, Germany; National Sun Yat-sen University, TAIWAN

## Abstract

Activist groups attack animal research and put scientists and their institutions under pressure, whereas scientists often remain silent. We report an interdisciplinary research project driven by a communication science perspective on how citizens respond to news reports about animal research (3 experiments, overall *N* = 765) and a German science-initiated information platform (“Tierversuche verstehen”; controlled user study, *N* = 100). Findings demonstrate that a critical journalist perspective within neutral, two-sided news reports (e.g., skeptical expert statements or images of suffering animals) does not affect citizen opinion strongly. Information media provided by scientific institutions seem to be welcomed even by citizens who hold critical prior attitudes. From these results, we develop a set of recommendations for future public communication of animal research that builds on best practices in organizational and crisis communication. These suggestions are intended to empower animal researchers to actively participate in public debate to support citizens’ informed attitude formation.

## Introduction

Animal research is a critically and emotionally discussed scientific topic at the science-society interface. One the one side of the controversy, activist groups in various countries seem to apply strategies to attract media attention and political support for their goal to limit or even ban scientific animal research [1–3]. Activist groups typically arrange protest events in which they express their moral condemnation of animal suffering caused by scientific studies. Their strategies aim at mobilizing public outrage by framing scientific studies of animals as cruel and unnecessary. Protest strategies often use images that suggest that physical harm is done to research animals [4] or publicly blame individual researchers for immoral or even illegal activities in their research [2, 5]. For example, in 2014, a German activist group publicly attacked a senior animal researcher at the University of Bremen by publishing full-page advertisements in several large newspapers that accused him of continuous cruelties against animals without any moral or scientific justification.

On the other side of the controversy, for many researchers in veterinary medicine, the life sciences, and biomedical research, the experience of being involved in a public controversy, the pressure to justify the fundamentals of their work, and the reception of allegations are new, irritating, and stressful. In this situation, many scientists and their institutions preferred to maintain silent and not to respond to criticism. It is beyond the scope of the present contribution to analyze why animal researchers have oftentimes decided against speaking out in the public [see 6]. From a communication science view on public discourse and publicized conflict, however, maintaining silence is a risky strategy, because it prevents citizens and political decision-makers to make informed judgments as they might not become aware of arguments for the necessity of animal research such as scientific progress [2].

The present article addresses the debate about animal research as one of many examples of an emotionally heated public controversy [7] focusing how the communication efforts of activist groups, media and science influence the attitude formation of the public. From a communication science perspective, we provide (1) a media effects approach examining how media portrayals influence laypersons’ attitude formation about animal research and (2) a science communication perspective on how scientific efforts to explain research procedures and requirements are assessed by the public. Our results on media effects are based on three social-scientific experiments on how news readers form attitudes and judgments. The experiments consider various samples of participants and different news media framings of animal research to replicate the findings and enrich the evidence base on attitude formation. Focusing on scientific communication efforts, we consider the effects of the website “Tierversuche verstehen” (German for “understanding animal research”) established by scientific institutions. The information service is intending to offer transparency and two-sided information on animal research. Among other things, the website publishes statistics on animal experiments, provides information on the conditions in which animals are kept, and describes the procedures used and their benefits, as well as alternative methods.

From this extensive line of inquiry, we develop conceptual conclusions on conflicts as a relevant dimension of the science-society interface as well as an applied perspective on the *public understanding of animal research*. We explicate the latter as practical recommendations on how the involved academic community should communicate about animal research methods to achieve a more balanced discourse and informed opinion formation by citizens in the future of animal research.

## Study 1 to 3: Media effects perspective

### News reports effects on citizens’ attitude formation

We adopt the concept of framing [8] from communication science to theorize how and why news media coverage may affect audience judgments of scientific animal research, covering global acceptance of animal research [e.g., 9] and four attitudinal facets that were derived from the analysis of past research, activist protest communication, and exchange with animal researchers: The perceived necessity of animal research, the assessment of animal research as morally justified, the level of support for stricter policies regulating animal research, and the degree of emotional concerns over the current practice of animal research and the suffering of laboratory animals.

The basic premise of explaining the persuasive framing effects of news media on various attitudinal facets is that audiences depend strongly on media information about animal research, because most people do not have access to first-hand experiences of animal research in their environment. For most citizens, the issue of animal research is therefore abstract and distal, so it is plausible to assume that many citizens do not hold strong opinions or much knowledge about it. Hence, most citizens will (have to) rely on media portrayals for orientation and judgment-forming about the value and legitimacy of animal research. In contrast, those citizens who already hold an attitude towards animal research are unlikely to be affected strongly by the media portrayal, because people tend to shield their existing opinions and to resist the persuasive influence of counter-attitudinal information [e.g., 10].

However, the elaboration likelihood model by Petty and Cacioppo [9] suggests that some influence on citizen opinion can be expected both for audience members who hold and those who do not hold a formed attitude if media reports include easy-to-process elements (so-called „peripheral cues“) that insinuate a certain position [9]. Such peripheral cues can trigger affective responses or evoke a quick cognitive impression of plausibility and validity. Among the content elements in news messages that affect audience judgements about animal research we aim to examine the role of expert statements, images, and framing of animal research as scandalous.

First, expert statements have been often used in journalism to secure information richness, validity, and credibility of their reports [e.g., 11]. For news audiences, experts serve as trustworthy, knowledgeable sources who are preferred over other sources (e.g., laypeople or openly partisan sources) for judgment forming [e.g., 12]. Hence, if journalists decide to state an expert who articulates a position towards animal research, her/his expert status may function as a cue to the audience that may shift their attitudes into the insinuated direction. If such effects occur, they are, according to the ELM, unlikely to manifest after single exposure [9]. However, small attitude shifts caused by one news report may be substantiated if citizens receive multiple similar messages over time.

Second, focusing on images accompanying news reports, it is assumed that they expand information richness, boost the interest value and audience appeal of the message, and trigger (strong) emotional reactions in news audiences oftentimes influential on subsequent attitude forming [e.g., 13–15]. In the present research context, publishing images of suffering animals is likely to serve as a peripheral cue that can shape audience judgments towards animal research through an emotional mechanism [e.g., 16]: Negative emotions triggered by the image (sadness, anger, outrage, or disgust) may fuel more negative attitudes towards animal research such as stronger support for banning or political restriction of animal experiments [17].

Third, the framing of animal research as scandalous may represent an important peripheral cue in news coverage. Journalists consider discovering scandals (and labeling events as scandals) as fundamental to their social role as watchdogs [5], and audiences seem to be attracted by the emotional appeal (e.g., a suspenseful story like a crime drama) that scandalization oftentimes entails [e.g., 18]). Moreover, scandalization in news may motivate audiences to call for far-reaching public policy responses to end the embarrassing or morally condemnable circumstances reported by the news [19]. Activists who oppose animal research try to put scandalized incidents on the news media’s agenda, for instance, public accusations of illegal housing conditions at scientific institutions of animal research or alleged violations of research restrictions imposed by authorities [e.g., 2]. Understanding how the general audience will respond to scandalizing news reports about animal research is therefore important to characterize the public discourse dynamics at the interface of science, politics, and society.

Based on the outlined rationale, the perspective on media effects in the present research program leads to the assumption of potential effects of three news message elements (expert statement, image of suffering animals, and scandal framing) on five relevant attitudinal facets as outcome parameters (audiences’ global acceptance of animal research and the four attitude facets explicated above). Consequently, the following hypotheses were derived:

Lower global acceptance, (b) lower perceived necessity, (c) lower perceived moral justification, (d) greater support for restriction policies, and greater emotional concern will be caused by

**H1:** … a news message presenting an expert statement that is critical of animal research compared to a news message presenting an expert statement that is supportive of animal research.
**H2:** … a news message including an image of a suffering (treated) animal compared to a news message including a visual of a healthy animal.
**H3:** … a news message framing the activities of animal research facilities as scandalous compared to a news message that does not contain a scandal frame of animal research facilities.

### Method

#### Overview

A series of three psychological experiments was conducted to illuminate the media effects perspective within the current research program. We conducted three experimental studies to replicate the findings within different samples, continuously revise the stimulus materials and consider further experimental conditions (see Table 1 for a comparison of the study design). In each experiment, participants were randomly assigned to read one news article about scientific animal research. Articles shown to participants had been manipulated systematically to either include or exclude one or several of those elements that had been theorized as potentially influential peripheral cues for audience judgment-forming (H1 to H3). For instance, in study 1, the article would cite an expert on animal research who would either announce that animal research “is not necessary anymore” or confirm that animal research is “still necessary” (i.e., manipulation of the presence versus absence of a critical expert statement, see H1). After reading the news article, participants were requested to respond to a questionnaire that included items to measure those attitude facets that were deemed conceptually relevant (global acceptance; perceived necessity, moral justification, support of stricter policies, and emotional concern; see above). Hence, comparing average scores of participants who had been exposed to different versions of the news report allowed tracing possible effects of the manipulated content elements (e.g., a critical expert statement) on citizen attitudes. While the types of peripheral cues that news articles featured differed between experiments, the overall research architecture was held constant across the three studies to improve robustness and generalizability through (partial) replication.

**Table 1:** Overview of media effects studies and manipulated content elements.

	Experiment No. 1	Experiment No. 2	Experiment No. 3
Manipulation of Expert Statement	X	X	X
Manipulation of Animal Visual	X	X	
	(suffering vs. healthy rabbit)	(suffering vs. healthy x rat vs. dog)	
Manipulation of Scandal Framing			X
Resulting Number of Experimental Groups	2x2	2x2x2	2x2
Number of Participants (N)	103	434	228

Table 1 provides an overview of the experimental studies by juxtaposing each experiment’s specific research design.

#### Experimental news articles on animal research

In all studies, the stimulus material consisted of a fictitious online news article discussing the necessity and relevance of animal research for medical research. It contained a fully balanced argumentation, as it addressed the same amount of arguments pro and contra animal research. To ensure external validity, all arguments stated for or against the necessity of animal research were derived from original material of both scientists and animal rights organizations. In addition, the article was embedded in the typical layout of a popular German online news site and included a central picture placed after the first paragraphs. Its visual appearance resembled real online news media, so the external validity of the stimulus message was high.

Within this article, the content elements of conceptual interest (expert statement, animal image, and scandal framing) were manipulated to create text versions in which a given element either suggested a rather critical stance towards animal research or a rather supportive stance (the stimulus material and images used in the study can be requested from the authors). However, it is important to stress that all article versions–regardless of a manipulated emphasis of either a critical or a supportive perspective–presented a mix of pro and contra arguments and hence resembled quality news reporting that follows professional-journalist norms of neutrality and two-sidedness. Thus, the studies simulated real news content and did not test the persuasive effects of a one-sided campaign or advertising messages that activist groups would typically disseminate.

The content element *expert statement* (H1) was manipulated in all three experiments (see Table 1). The stimulus article cited a fictitious expert who was introduced as a professor of medical ethics. In one article version, his statement was critical of animal research, as he declared it unnecessary due to the availability of alternative methods in medical research. In the alternative article version, the expert statement characterized animal research as still necessary due to the unavailability of sufficiently functional alternatives. To ensure that participants would notice the statement, it occurred not only in the article text but was also quoted in the headline of the article.

The content element of the *animal image* (H2) was manipulated in studies 1 and 2. In study 1, a photo that was attached to the news article either showed a white rabbit that had undergone an experiment as it was partially shaved and displayed stitched injuries. In the alternative condition, the photo showed an ostensibly healthy white rabbit without any notable cues of impaired health, pain, or previous treatment. In study 2, the same type of manipulation (photos of suffering versus healthy animal) was implemented; however, instead of a white rabbit, two other species were depicted: One set of images presented a (suffering versus healthy) dog; the second set of images displayed a (suffering versus healthy) rat. The rationale behind this more nuanced manipulation of animal imaging in the news article was that past research had found greater public acceptance for animal research involving pest species (such as mice or rats) and lower tolerance for research with species commonly known as pets (such as cats or dogs) [20].

Finally, the content element of scandal framing (H3) was manipulated only in study 3. For this manipulation, a paragraph was added at the beginning of the article on a police investigation against a fictitious animal testing facility in Germany where the conditions of keeping were denounced. The prevailing circumstances and the handling of the animals were explicitly described as a public scandal. In the alternative condition, this paragraph was not included.

#### Measurement of attitude components in news readers

Self-report Likert-type scales were applied in all studies to measure those attitude components that had been conceptualized as potentially affected by news framing of animal research (see hypotheses section). First, as a global measure of the opinion towards animal research, respondents of all studies rated their general level of acceptance of animal research by a single item. They rated their agreement to the statement “It is acceptable that there is animal research for medical purposes” on a five-point Likert-type scale ranging from 1 (do not agree at all) to 5 (fully agree).

To account for attitudes towards animal research in more detail, we differentiated between the perceived necessity, moral justification, support for stricter policies, and emotional concerns capturing the typical lines of arguments which can be found in the debate about animal research. Throughout the three experiments, the individual items that measured these dimensions were optimized, supplemented, and partially adapted to the stimulus material. S1 Table in S1 File gives an overview of the items used in each study. While the dimensions of perceived necessity, moral justification, and emotional concerns were only slightly modified, the policy support dimension was fundamentally revised for the third experiment. These extensive changes appeared necessary to better relate to the scandalization of keeping conditions of animals addressed in the stimulus material. All items were measured on five-point Likert-type scales and combined into mean indices for further analyses. All indices ranged between acceptable and very good reliability (S1 Table in S1 File).

#### Samples and procedures of experiments

To experimentally examine audience responses to different news framing of animal research, the effect of message properties (but not person characteristics) was central to the present studies. Consequently, the experiments were set up as a replication series with similar stimulus messages, but different samples, aiming for high generalizability across heterogenous demographics.

The first experiment was conducted with *N* = 103 students that aged between 18 and 46 years (*M*_age_ = 21.34; *SD*_age_ = 3.76; 44% females). The student sample provides an efficient way of an initial exploration of news effects on attitude formation. Participants were recruited in three university courses of public relations, life science, and electrical engineering programs of universities in a major German city. The experiment was conducted during regular class sessions.

The second (*N* = 434) and third (*N* = 228) experiments were conducted with German individuals from diverse demographic backgrounds to cover different age groups and educational levels, which might be relevant for attitude formation. In both studies, the participants were recruited by a large, trained student team who approached family members, friends, colleagues, and weak tie contacts. To ensure heterogeneity, recruiters were instructed to meet quota requirements for age groups, gender, and educational level. This way, robust generalizability of findings about message effects was ensured. However, full representativeness of the German population was impossible to achieve with the current approach and available resources.

In the second study, participants were aged between 18 and 87 with a mean age of *M* = 39.26 years (*SD* = 17.96). About 54 percent of the respondents were female. Participants’ educational backgrounds varied substantially, ranging from basic secondary school certificate (15%) to ordinary level (27%) to high school diploma (33%) to graduation from university (32%).

The participants in the third study aged between 18 and 87 years, but the mean age was lower with *M* = 32.46 (*SD* = 14.27). About 53.5 percent of the respondents were female. The educational background ranged from basic to secondary school certificate (28.9%) to high school diploma (44.4%) to graduation from university (26.7%).

In all studies, the participants were randomly assigned to one of the experimental conditions and received a paper-pencil questionnaire that included a printout of the stimulus news article. At the beginning the participants of each study were comprehensively informed about the scientific purpose of the study, their right to decline to participate and to withdraw from the research once participation has begun, the confidentiality of their responses, and whom to contact for any questions concerning the study. After reading the study description, informed consent was provided by all participants by signing the first page of the paper-pencil questionnaire. To protect the privacy of our participants, the personally identifiable information was stored separate from the collected survey data. After answering initial questions, participants were asked to read the printed news article. Subsequently, evaluations of the article and attitudes towards animal research were assessed. Finally, participants were thanked and debriefed.

To ensure successful randomization, we tested for unintentional group differences regarding socio-demographics, pet ownership, and membership in animal rights NGOs. None of the three studies showed significant differences between the experimental groups. Besides, we ran a treatment check for each study, providing the credibility and authenticity of our stimulus material. All articles are considered as rather interesting, typical, and credible (see Table 2). Again, there are no significant differences in the comparison between the individual experimental conditions.

**Table 2:** Results of the treatment check.

	Experiment No. 1	Experiment No. 2	Experiment No. 3
	*M* (*SD*)	*M* (*SD*)	*M* (*SD*)
Interesting	3.42	3.80	3.69
	(.97)	(1.03)	(1.04)
Typical	3.33	3.25	2.90
	(1.02)	(1.37)	(1.10)
Credible	3.55	3.73	3.74
	(.94)	(.88)	.91

*Note*. Scale from 1 = “do not agree at all” to 5 “agree completely”

### Results

#### Descriptive analysis

First, descriptive analyses showed across all studies that respondents have a balanced attitude towards animal research. In general, the respondents considered animal research for medical purposes as rather acceptable, necessary, and partly morally justifiable (see Table 3). The support for stricter regulation policies varied considerably between studies but was rather limited. The discrepancies between the first and second compared to the third study can be attributed to the revision of the measurement in experiment 3. Emotional concerns turned out relatively low in the first and third experiment but were found substantially higher in the second study (see Table 3).

**Table 3:** Overview of the attitudes towards animal research.

	Experiment No. 1	Experiment No. 2	Experiment No. 3
	*M* (*SD*)	*M* (*SD*)	*M* (*SD*)
Global acceptance	3.5	3.21	3.31
	(1.15)	(1.37)	(1.22)
Necessity	3.62	3.18	3.41
	(.79)	(.96)	(1.05)
Moral justification	3.29	2.87	3.03
	(1.01)	(1.08)	(1.12)
Policy support	3.33	3.82	2.32
	(.76)	(.72)	(.85)
Emotional concerns	2.50	3.65	2.30
	(1.36)	(1.10)	(1.24)

*Note*. Scale from 1 = “do not agree at all” to 5 “agree completely”

#### Effects of critical news framing on citizen attitudes towards animal research

Analyses of variance were conducted for each experiment’s data set to test the hypotheses. H1a-f on the effect of critical expert statements on citizens’ attitudes was examined using data from all three experimental studies. All studies revealed the same descriptive trend that readers reported global acceptance and specific attitudes that were slightly more congruent with the position insinuated by the expert statement. However, most of the effects were non-significant or were very weak; concerning the global acceptance measure, the observed effect size was rather low in study 1 (partial η^2^ = .07), very small in experiment 2 (partial η^2^ = .02) and close to zero in experiment 3 (partial η^2^ = .01). Similar effect sizes ranging between zero and .05 were observed for the measures of more specific attitude components. Hence, the repeated test of H1 with three different samples returned no or only very weak support (see Table 4); in general, citizens did not respond to critical expert statements by shifting their (pre-existing or newly formed) attitudes towards animal research. Table 4 provides an overview of all group comparisons on expert statement effects across the experiments. Details on the single effects examined in each experiment can be found in the see S2 to S10 Tables in S1 File.

**Table 4:** Overview of the test of Hypothesis 1: Effects of the expert statement on acceptance and attitudes towards animal research.

	Experiment No. 1	Experiment No. 2	Experiment No. 3
A critical compared to a supportive expert statement will cause …			
H1a: lower global acceptance	Accepted	Accepted	Rejected
H1b: lower perceived necessity	Rejected	Rejected	Rejected
H1c: lower perceived moral justification	Accepted	Rejected	Rejected
H1d: greater support for restriction policies	Accepted	Rejected	Rejected
H1e: greater emotional concern	Rejected	Rejected	Rejected

*Note*. With a significance level of p < 0.05, a hypothesis is considered as accepted.

Data from experiments 1 and 2 were used to test H2a-e on the effects of animal visuals in news reports on readers’ attitudes (see Table 5 for an overview as well as S1-S10 Tables in S1 File). The findings showed that the news audience did not develop systematically lower acceptance of animal research after exposure to images of suffering (versus healthy) animals that accompany a news report. In experiment 1, greater emotional concern was triggered by the image of the treated rabbit (compared to the image of the healthy rabbit, partial η^2^ = .04); however, this small effect was not replicated in experiment 2 (see Table 5). Hence, H2 was disconfirmed by the data, as the news audience was not found overly responsive to images of (treated versus healthy) laboratory animals.

**Table 5:** Overview of the test of Hypothesis 2: Effects of the animal images on acceptance and attitudes towards animal research.

	Experiment No. 1	Experiment No. 2	Experiment No. 3
A news message including an image of suffering (treated) animal will cause …			
H1a: lower global acceptance	Rejected	Rejected	-
H1b: lower perceived necessity	Rejected	Rejected	-
H1c: lower perceived moral justification	Rejected	Rejected	-
H1d: greater support for restriction policies	Rejected	Rejected	-
H1e: greater emotional concern	Accepted	Rejected	-

*Note*. With a significance level of p < 0.05, a hypothesis is considered as accepted.

Finally, data from experiment 3 served to examine H3a-e on the effects of scandal framing in news reports on readers’ attitudes towards animal research. In this study, no effect of a scandal frame on global acceptance or specific attitudes towards animal research occurred (see S8-S10 Tables in S1 File). Hence, H3a-e were rejected.

Focusing the combinations of cues, the findings indicate short-term effects even after reception of a single news article: For instance, in study 2, general acceptance scores of news readers who had seen an article that contained a pro-research (uncritical) expert statement plus the image of a healthy rat were notably higher (*M* = 3.41, *SD* = 0.83) than the general acceptance of participants who read an article version that cited a critical expert statement and presented an image of a treated dog (*M* = 2.94, *SD* = 0.94). Thus, for this comparison of experimental groups who had either been exposed to a news message that lacked any critical cues or to an article with two combined skeptical frame elements (expert statement and animal image), a substantial discrepancy in resulting acceptance emerged (Cohen’s *d* = 0.53, a medium-sized effect, see [21]).

### Discussion

Our extensive experimental research program on news audiences’ response to news articles animal research through different cues (expert statement, animal image, scandal framing) demonstrates that people are *not* susceptible to strong short-term persuasion by news reporting. Readers do perceive and react to negative cues, and the inclusion of multiple critical frame elements results in notably lower acceptance for animal research. However, a scenario of news articles that contain combined negative framing elements is very rare in the real news landscape, because most articles are likely to contain balanced pro- and contra arguments, and hence no one-sided over-representation of negative critical cues. An exception to this rule applies to tabloid newspapers that aim for strong affective activation of their audiences and tend to sacrifice journalistic balance for this purpose (see general discussion below).

The small or even non-existent effects can be explained by two causes. First, those individuals who are only weakly involved with animal research (that is, hold little knowledge and no strong opinion about it) may not have found the experimental news article to suggest one specific position. Because they lack background knowledge (or interest), they might not have recognized that certain elements in the article signal a certain stance towards animal research. According to the ELM, low-involvement readers are likely to overlook such message details, as they would be unable to judge the relative importance or quality of stated arguments. Consequently, low-involvement participants may not have been responsive to (negative) news framing of animal research, as they did not recognize the position suggested by the news article. A second plausible cause is people’s proclivity to shield their existing attitudes against persuasion [9]. For shielding pre-existing attitudes, readers will probably utilize balanced content elements, as they find some information that helps bolster their opinion even if the overall article tendency would contradict it. Quality news reporting that contains two-sided coverage will thus limit its own persuasive power. To the extent that citizens already hold a view on animal research, they are unlikely to shift that view substantially after a confrontation with a single (high-quality) news item.

Also images of suffering animals do not drive citizens’ resentment through negative emotions. Slightly increased emotional concern after exposure did not feed forward into a strikingly greater rejection of animal research. Concerns about the potential influential power of animal images thus seems to be exaggerated. While people respond to images of (cute) animals who suffer and this response will certainly motivate some public outrage in protest campaigns that use such images [15], news coverage that presents a balanced, two-sided view does not turn into powerful anti-science persuasion only because it comes with an image of a treated animal.

So, if the peripheral cues of news articles do not massively affect citizens’ self-reported attitudes towards animal research, what does influence them instead? Past research in science communication suggests that pre-existing attitudes or general values for those individuals without preexisting views play the most important role [e.g., religious beliefs: 22]. Political orientations and ideological beliefs about human-animal relations are important [23]. For younger generations, the protection of nature and animals has been observed as a consensual mainstream value orientation that drives, among others, vegetarian or vegan nutrition behavior [24]. So, while single news media articles do not influence audience positions towards animal research substantially, long-term dispositions such as value orientations probably rule these positions. These assumptions suggest distinguishing between subgroups and consider long-term dispositions such as age and values. One particularly important person characteristic that follow-up research should also address is science literacy [25]. People differ tremendously in the extent to which they are educated in how science works and how they could form a reflected opinion about (controversial) scientific issues. Such differences are likely to influence opinion formation also in the present context. Assessing the (presumably moderating) effect of science literacy in citizens’ evaluation of animal research and its public debate is therefore key to improve our understanding of the science-society interface in this special case

An alternative explanation for small and non-effects observed in the present study is a pragmatic reasoning of the public. Scientists in general enjoy an image of benevolence, as they engage in improving the health conditions of the population and strive for patient safety and innovation in healing [26, 27]. In this vein, animal research for which a (distal or even direct) medical utility can be claimed (which applies to much animal research) is likely to be accepted by many people, as they apply a trade-off calculation of problems (e.g., harm to animals) and advantages (e.g., improved medical treatments in the future). Thus, they register and understand negative information in news reports, yet they still apply a cost-benefit calculation that will oftentimes result in non-persuasion from critical news messages [28].

## Study 4—science intervention perspective

### Citizen’s evaluation of and reaction to science communication

Given an observable decline of science journalism [29–31], communication efforts by scientific institutions and scientists to reach out to the public are on the rise and hence deserve consideration in the present context. Science communication efforts on animal research aim to increase the public *understanding* of (animal) science, and to promote public acceptance through transparency of ethical principles, demonstration of efforts to serve the well-being of animals (3R principles), and constructive response to public criticism [32]. One example of such public communication about animal research is the German initiative “Tierversuche verstehen” (“Understanding animal experiments”). Funded by the German Alliance of Scientific Organizations (“Allianz der Wissenschaftsorganisationen”), this online platform aims to cover multiple aspects of animal research and its public discussion in a journalism-like fashion.

The current study aims to focus the citizens’ perspective on those activities. If audiences would judge science communication messages about animal research as not credible, low-quality, or low-value, such communication initiatives would fail to improve the science-society dialogue and the promotion of understanding and acceptance of animal research. Even boomerang effects might occur in the sense that negatively judged science communication would backfire and increase citizens’ resentment [33]. Hence, study 4 served to provide insights on audience responses to science communication about animal research, focusing on “Tierversuche verstehen” as the case of investigation. We developed the following research question to examine how the public evaluates public communication about animal research originated by scientific initiatives such as “Tierversuche verstehen”:

**RQ1**: How do citizens judge the quality, credibility, and overall value of communication about animal research such as the scientific initiative “Tierversuche verstehen”?

The second aim is to examine the audience perception referring to hostile media perceptions, which is a social phenomenon discovered in past research on publicized conflicts [34]: Depending on their attitude towards a conflict-loaden issue, audience members tend to judge two-sided, neutral news coverage of the issue as biased against their attitudes. This applies to people who identify with *any* of the parties. Motivated reasoning and self-categorization processes have been theorized as psychological mechanisms underlying such biased judgments of news messages [e.g., 35].

This phenomenon is particularly relevant to consider in the case of science communication that try to achieve balance and two-sidedness. As the respondents know that the message has been originated by scientists and not by (neutral) journalists, it might be more likely that citizens who hold critical attitudes toward animal research ("opponents”) assume one-sidedness, omission of negative views about animal research, or even manipulative intent [34]. Citizens with neutral or supportive attitudes towards animal research ("proponents”), in contrast, are less likely to make such negative judgments. Hence, based on the rationale outlined above, we derived the following hypothesis about how attitudes towards animal research affect quality assessments and perceived biases of public science communication messages:

**H4**: Citizens who hold critical attitudes towards animal research (“opponents”) are likely to report (a) a lower quality and (b) a more pronounced bias of public science communication messages such as “Tierversuche verstehen” than citizens who hold neutral or supportive attitudes (“proponents”).

### Method

#### Participants and procedure

To investigate citizens’ perception and evaluation of scientific communication about animal research, we conducted a computer-assisted personal survey. A local market research institute recruited a demographically diverse sample of 100 individuals (51% female, age: 19–64 years; *M* = 44; *SD* = 14.45; 64% with at least a secondary school certificate) in a major German city. The commercial market research company that recruited the participants and collected the data for Study 4 is certified in accordance with ISO norm 26362 and follows the ICC/ESOMAR International Code on Market, Opinion and Social Research. In the beginning, participants were informed about the purpose of the study, their right to decline to participate in the study once participation has begun, and the confidentiality of their responses. After reading the study description, informed consent was provided by all participants by clicking the “start” button to access the survey. At first, the participants answered a short questionnaire about their initial attitudes on animal research, their involvement with the issue, and their identification with opponents or proponents of animal research [36]. Subsequently, respondents visited (for at least 15 minutes) the website “Tierversuche verstehen” (www.tierversuche-verstehen.de). Afterward, respondents answered questions about the perceived quality and bias of the website, perceived effects on different groups of people, and perceived threat to their social identity, all of which served to assess hostile media perceptions.

#### Measures

*Attitudes toward animal research* were assessed with seven items asking respondents about their general acceptance, perceived necessity, moral legitimacy, emotional concerns, and acceptance of animal research practice (e.g., “The thought of animals suffering for medical experiments burdens me.”, “It is acceptable that animal research is conducted for medical reasons.”). Items were combined into a mean index indicating general attitudes towards animal research. A semantic differential was used to measure *involvement* with the issue of animal research (e.g., “irrelevant–relevant”; “unimportant–important”). After respondents assigned themselves to the groups of opponents or proponents of animal research (“All in all, would you rather assign yourself to the group of proponents or opponents of animal experiments?”), *social identification* with the respective group was measured by adapting the self-investment dimension of the German version of the social identification scale to the context of animal testing (e.g., “It is pleasant to be an opponent of animal research”; 39). Two indices were computed representing the identification of opponents and proponents with their respective groups.

Drawing on Brosius and Birk [37], the *quality assessment* of the information website was measured with eleven items (e.g., “credible”, “balanced”). Following previous studies on hostile media perception [e.g., 38], *website bias* was measured with four items assessing the tendency towards proponents’ point of view (e.g., “The authors of the website are probably rather proponents of animal experiments”). Table 6 displays the means, standard deviations, and reliabilities of the measures.

**Table 6:** Means, standard deviations, and reliabilities (Cronbach’s Alpha) of measures.

	*M*	*SD*	α
Attitude towards animal research	3.07	.84	.81
Involvement	3.85	.87	.90
Social identification opponents	3.24	.84	.89
Social identification proponents	2.63	.83	.90
Quality assessment of “Tierversuche verstehen”	3.59	.89	.86
Perceived bias	3.74	.85	.83

### Results

Descriptive analyses show that overall respondents have a balanced attitude towards animal research (*M* = 3.07; *SD* = .84), although 66 percent identified themselves as opponents rather than proponents (Table 6). Comparing the two opposing groups, opponents identify more strongly with their group than proponents (t(98) = 3.468; p < .001; *η^2^* = .109). The respondents seem to be somewhat involved with the issue (*M* = 3.85; *SD* = .87). Concerning the website (cf. RQ1), the participants attribute a relatively high quality to the content (*M* = 3.59 *SD* = .89), although they perceive the website to be slightly biased towards a more positive view on animal research (*M* = 3.74; *SD* = .85).

To identify differences in the perception and evaluation of opponents and proponents, we conducted ANOVAs(H4). Results showed that the groups differ in the evaluation of the quality (H4a) but did not differ in the perception of bias (H4b) (see Table 7). Proponents of animal research evaluated the quality of the website as slightly better than opponents. A closer look at the individual items measuring quality assessment revealed significant differences between groups regarding evaluations of the balance, comprehensiveness, ethical acceptability, usefulness for society, the article being a waste of time, and a pleasant experience. The effect sizes with which the observed differences materialize were relatively small, which indicates that the website “Tierversuche verstehen” receives relatively positive judgments from citizens with differing attitudes towards animal research. The two-sided approach of the science communication initiative thus seems to succeed in offering believable information and avoiding strongly polarized audience responses (i.e., hostile media perceptions among citizens with critical prior attitudes).

**Table 7:** Means, standard deviations, and ANOVA results for quality assessment and perceived bias for opponents and proponents.

	Opponents	Proponents				
	*n* = 66	*n* = 34				* *
* *	*M (SD)*	*M (SD)*	*df*	*F*	*p*	*eta* ^2^
Quality Assessment	3.43 (.87)	3.89 (.85)	1	6.407	.013*	.061
Interesting	3.67 (1.23)	4.12 (1.09)	1	3.236	.075	.032
Balanced	2.91 (1.11)	3.47 (1.08)	1	5.879	.017*	.057
Comprehensive	3.64 (1.19)	4.12 (.95)	1	4.217	.043*	.041
Credible	3.27 (1.14)	3.74 (1.02)	1	3.931	.050	.039
Illustrative	3.56 (1.14)	3.76 (1.13)	1	.725	.397	.007
Well written	3.52 (1.17)	3.76 (1.02)	1	1.117	.293	.011
Ethically acceptable	3.08 (1.10)	3.88 (.84)	1	14.006	.000*	.125
Of high quality	3.50 (1.11)	3.65 (1.18)	1	.377	.541	.004
Useful for society	3.53 (1.10)	4.09 (1.06)	1	5.944	.017*	.057
Waste of time	2.15 (1.26)	1.53 (1.05)	1	6.125	.015*	.059
Pleasant experience	3.23 (1.12)	3.76 (.99)	1	5.586	.000*	.054
Perceived Bias	3.72 (.87)	3.77 (.82)	1	.073	.787	.001

*Note*. Univariate ANOVAs; dependent variables measured on 5-point Likert-type scales (1 “does not apply at all” to 5 “does apply completely”

*p < .05

### Discussion

The investigation of audience judgments revealed that “Tierversuche verstehen”received a positive average evaluation of quality and credibility and did not cause severe hostile media perceptions, which is one precondition for an informed judgements about animal research [33]. Regarding message design, the two-sided, quasi-journalistic approach that “Tierversuche verstehen” has developed results in positive audience reactions and thus serves as a valuable instrument to offer balanced expertise and scientific views on the public debate. The efforts of the initiative appear to be a good investment in making science perspectives visible within the public debate, providing expert information, and promoting informed judgment forming by citizens concerning the public controversy over animal research.

The present study should, however, not be misinterpreted about the magnitude of impact that even highly professional messages of science communication such as “Tierversuche verstehen” can achieve. Most citizens will never search actively for information about animal research, as the personal relevance of the topic is mostly low in everyday life. Without expensive investments in visibility and advertising, information websites such as “Tierversuche verstehen” will not succeed in reaching out to many citizens or large segments of society. In this sense, other and additional measures of science communication are important (see general discussion section below). However, for those citizens who find animal research relevant the presence of a well-designed, audience-friendly information platform with a two-sided communication approach is highly important, because highly involved people are more likely to find and consume science communication messages, and communicating to and with them will make a most important difference for articulating science perspectives on animal research in the public debate.

Moreover, while only a minority of citizens will develop substantial involvement with animal research, the utility of information initiatives such as “Tierversuche verstehen” will also depend on citizens’ science literacy [25]. Understanding the controversy around animal research requires an advanced level of scientific literacy. Future research should therefore consider (and measure) participants’ science literacy as potential moderator of science communication’s effects; beyond the research perspective, science communicators could and should understand information services such as “Tierversuche verstehen” as chance to contribute to exactly the science literacy that societies need to develop in their populations.

## General discussion and recommendations to animal researchers

### Summary of empirical findings

The present research program has addressed how communication strategies used on in the public controversy about animal research influence public attitude formation. Our experimental audience studies revealed that the impact of expert statement, image, and scandal framing on the general acceptance of animal research is weak. This finding is in line with past research on media persuasion which rarely found massive attitudinal effects of single messages [9] and suggests that animal researchers should not anticipate far-reaching consequences if they find a (seemingly) critical report about their profession in the news. Finally, an exploratory audience study with an online information platform about animal research (“Tierversuche verstehen”) revealed generally benevolent judgments of citizens; the mere presence of the platform, but also the quality of two-sided portrayals of animal research were found informative and credible among individuals who held supportive, neutral, and critical attitudes towards animal research.

### Conceptual implications

All branches of science strongly depend on public acceptance and trust. While the insights that researchers achieve and their applications continue to demonstrate the value of a free, independent, and thriving science, the growing importance of science in increasingly complex and diverse societies have also turned scientific institutions and agents into participants, stakeholders, and sometimes targets of public discourse and controversy (e.g. [39, 40]). Some of these controversies about science, its methods, and results have been ignited by interest groups who pursue instrumental goals such as preventing climate protection politics [41]; other controversies relate to inherent normative challenges of science and technology, such as in genetically modified organisms [e.g., 42]. The public controversy over animal research has emerged among these ‘conflict zones’ at the science-society-interface already long ago [e.g., 15]. Continued activity of protest groups keeps the controversy alive and seems to have intensified in some countries recently [e.g., 2]. These ongoing protest dynamics occur within a broader social trend of increased public expectations towards science regarding transparency, active communication, and dialogue with media and citizens at eye level [43]: While many scholars continue to view lay audiences to lack knowledge and understanding of science [(„deficit model“, cf. 44], many political decision-makers, journalists, and citizens are not willing to grant unquestioned privilege and obligation-free support to science but insist on more academic transparency and humility instead. This potentially benign development materializes in, among many other phenomena, greater expectations towards animal researchers to contribute to the mitigation of the societal conflict over animal research, both through behavioral change (3R) and engagement in science communication.

Against this background, the present line of research has continued previous social science work on the public debate over animal research. The present studies do not reveal a drastic intensification of the public controversy over animal research, at least in the German context. News consumers were found to respond emotionally to images of treated animals [17] but to mostly resist persuasion effects towards less acceptance of animal research. We attribute the limited effects of critical coverage to a combination of news media’s two-sided mode of reporting, the robustness of pre-existing attitudes, and citizens’ pragmatic cost-benefit-considerations that emphasize the utility of animal research. We can thus portray the current state of animal research as a rather latent than acute conflict zone between science and society. Efforts of active science communication such as “Tierversuche verstehen” clearly contribute to the active dialogue and make the science perspective visible within the public debate; they thus represent important responses to the general call for more active engagement of science in dialogue with society. Positive judgments of quality and credibility obtained from empirical audience explorations such as the present study 4 confirm the value of such science communication platforms.

### Applied perspectives: Recommendations on science communication of animal research

The present work allows deriving applied recommendations for animal researchers and their academic institutions for their future engagement with the public controversy over their work.

#### Recommendation 1 –Stay calm about critical news, be alert about personal hostility

Animal researchers may notice critical news media coverage about their field. Stress is an understandable response to such coverage [45], and scholars may anticipate strong negative reactions in the public because of such news reporting. The present findings (studies 1 to 3) suggest that such worry is not justified, since single negative news reports are unlikely to shift public opinion. Constant vigilance for public coverage of animal research is certainly advisable, but alertness for negative media resonance is not.

This general recommendation does not apply, however, in the case of ad-hominem attacks put forth by activist groups. Victimization by radical protesters is a massive threat, and more frequent incidents of hostility against researchers have been recorded in recent years (e.g., [46]). Thus, accusations or personal threats from activists should be taken very seriously. Affected scholars should respond actively, involve their colleagues and research institution as well as authorities to protect themselves and build up solidarity in their community. Supervisors and university executives bear an important responsibility to provide organizational, material, and social support to animal researchers in such critical circumstances.

#### Recommendation 2 –End the silence, engage in public communication

Some researchers have developed a strategy of maintaining silence in a public arena. The present conclusion that many citizens, journalists, and political decision-makers are *not* hostile should motivate any animal researcher to actively engage in more public communication about their work such as the 3R principles of animal research.

Several positive consequences will arise from such engagement. First, interacting with many diverse citizens will result in a more balanced perception of how people perceive, valuate, and judge animal research. Second, non-scientific stakeholders will appreciate such communication efforts and experience animal researchers as responsible scientists who are willing to listen to public concerns, which could play out in greater trust [47]. The findings from study 4 support this prediction. Third, journalists will consider information provided by scientists for their news making, which may lead to increased media resonance and greater visibility of scientific views on animal research and hence, to better chances for citizens to develop well-informed opinions.

Institutional leaders are advised to strategically plan and enable public communication efforts to systematically overcome current tendencies of remaining silent. The institution should be able to speak publicly also about controversial aspects, but most importantly, it should demonstrate the willingness to listen to and interact with political decision-makers, journalists, and citizens. “Tierversuche verstehen”, the platform investigated in study 4, is an excellent example for this approach.

#### Recommendation 3 –Develop communication strategies for specific target audiences

Most science communication efforts seem to address (interested) citizens (e.g., websites or local events) but neglect political decision makers and regulation authorities as target audience. These actors can affect and constrain future research activities most directly through legislation, extension, or limitation of available funding. They oftentimes receive critical information from activist groups and may be more susceptible to negative media reports than the general population that was found only weakly responsive in studies 1 to 3 (“influence of presumed influence”, cf. [48]). Therefore, animal researchers and their institutions should also provide political leaders with compact documents that explain their positions, explain 3R principles, and indicate the merits and necessities of continued animal research for human health and patient safety. In this vein, political decision-makers should be well-aware of existing science communication activities such as “Tierversuche verstehen” (study 4). As a result, different target audiences would be empowered for well-informed opinion formation.

#### Recommendation 4 –Prepare for unforeseen events of crisis

The current findings of little susceptibility of news audiences to negative news framing (studies 1 to 3), and of positive citizen response to science communication efforts (study 4) need to be qualified by a perspective on crisis communication [49]. A crisis may occur in any institution involved in animal research, for instance, if accidental problems with housing conditions are leaked to the public and instrumentalized by active groups [7, 18] or tabloid news media. If such a crisis would occur, the currently rather ‚cool‘ conflict zone of animal research between science and society (as it materialized in the results of studies 1 to 3) would rapidly turn ‚hot‘.

The best prevention of such a crisis scenario is, of course, good scientific practice, effective implementation of 3R, and good management of research and housing facilities. Still, it is advisable for all institutions conducting animal research to prepare for unforeseen crisis events. Situational crisis communication theory [49] offers helpful insights for developing ideas on crisis readiness. While an in-depth elaboration of these insights is beyond the scope of the current work, we emphasize that researchers and executives can prevent much damage by anticipating potential crisis scenarios and planning how they intend to communicate with key target audiences (see recommendation 3). Maintaining silence is, as elaborated above, not advisable in normal times; it is the worst option to choose in times of crisis.

## Conclusion

The present contribution has reported a program of applied communication science to characterize the public controversy over animal research as a conflict zone of the science–society interface. From systematic empirical data on news effects and citizens_’_ responses to an online information platform of science communication, we can offer robust insights and develop advice for future public communication of animal research. The observation that the *conflict zone* is rather *cool* and that preconditions for effective science communication are promising despite continuous activist protests should be understood by animal researchers and their institutions as an opportunity to engage more actively in public communication efforts. The recommendations that we have developed synthesize learnings from past communication science on public relations, science communication, and crisis communication. They shall inform and enable animal researchers who often find it difficult to deal with the *perpetrator role* that activists and some news media seem to force on them and to hold their ground when confronted with normative allegations. We offer these recommendations to empower animal researchers to take an authentic stand in the public controversy, make scientific arguments and perspectives more visible, and contribute to a well-informed, rational discourse over the costs and benefits of animal research for science and society.

## Supporting information

S1 File Additional information about the studies and findings. (DOCX)
