# Supplementary material for: Public controversy and citizens’ attitude formation about animal research: A case for scholarship and recommendations on conflicts at the science-society interface
Source: PLoS One. 2024 Jan 3;19(1):e0295503. doi: 10.1371/journal.pone.0295503 (PMC10763933; doi:10.1371/journal.pone.0295503)
Supplement: S1 File — (DOCX) [file pone.0295503.s001.docx]

**Supporting information**

**S1 Table. Overview of Scales and Items used in Study 1-3**

| Used in which study (I/II/III) | | |  | Study 1 | | | | | Study 2 | | | | | | Study 3 | | | | | |  |
| --- | --- | --- | --- | --- | --- | --- | --- | --- | --- | --- | --- | --- | --- | --- | --- | --- | --- | --- | --- | --- | --- |
|  |  |  |  | M | SD | | α | | M | | SD | | α | | M | | SD | | α | |  |
| **Global Acceptance** | | | | **3.53** | **1.15** | | **-** | | **3.21** | | **1.37** | | **-** | | **3.31** | | **1.22** | | **-** | |  |
| **I – III** | | It is acceptable that there is animal research for medical purposes. | |  | |  | |  | |  | |  | |  | |  | | | | | |
| **Necessity** | | | | **3.62** | **0.79** | | **.798** | | **3.12** | | **0.89** | | **.845** | | **3.41** | | **1.05** | | **.868** | |  |
| **I – III** | There should only be animal research for life-threatening diseases.* | | |  |  | |  | |  | |  | |  | |  | | | | | |  |
|  | Animal research is not necessary anymore, as there are alternative methods. | | |  |  | |  | |  | |  | |  | |  | | | | | |  |
|  | Animal research is unjustified because the assignability to humans is questionable. | | |  |  | |  | |  | |  | |  | |  | | | | | |  |
|  | Medical animal research is important for securing medial progress. | | |  |  | |  | |  | |  | |  | |  | | | | | |  |
|  | I think it is important to test medicals with animals before applying them to humans. | | |  |  | |  | |  | |  | |  | |  | | | | | |  |
| **II** | The chance for cure of patients should be rated as more important than the protection of laboratory animals. | | |  |  | |  | |  | |  | |  | |  | | | | | |  |
|  | Hundreds of laboratory animals should not suffer for curing a rare disease or to help only a small group of patients. | | |  |  | |  | |  | |  | |  | |  | | | | | |  |
| **Moral Justification** | | | | **3.29** | **1.01** | | **.864** | | **2.87** | | **1.08** | | **.908** | | **3.03** | | **1.12** | | **.889** | |  |
| **I – III** | All experiments not to be conducted with humans should also not be conducted with animals. | | |  |  | |  | |  | |  | |  | |  | | | | | |  |
|  | Medical animal research is ethically justifiable. | | |  |  | |  | |  | |  | |  | |  | | | | | |  |
|  | Because of the medical benefit animal research is morally legitimated. | | |  |  | |  | |  | |  | |  | |  | | | | | |  |
| **I + II** | Animals should not be used for medical research, as they cannot advocate their interests. | | |  |  | |  | |  | |  | |  | |  | | | | | |  |
| **II** | Scientists, who carry out medical tests with animals, act in an ethical manner. | | |  |  | |  | |  | |  | |  | |  | | | | | |  |
|  | Scientists, who carry out medical tests with animals, are credible. | | |  |  | |  | |  | |  | |  | |  | | | | | |  |
| **II + III** | In my opinion, medical animal research is morally shameful. | | |  |  | |  | |  | |  | |  | |  | | | | | |  |
| **Policy Support** | | | | **3.33** | **0.76** | | **.641** | | **3.82** | | **0.72** | | **.620** | | **2.32** | | **.85** | | **.70** | |  |
| **I + II** | The administration should supervise animal research strictly and regularly. | | |  |  | |  | |  | |  | |  | |  | | | | | |  |
|  | Contraventions should be punished more often. | | |  |  | |  | |  | |  | |  | |  | | | | | |  |
|  | All medical animal research should be forbidden by law. | | |  |  | |  | |  | |  | |  | |  | | | | | |  |
|  | All medical animal research that causes pain or injuries should be forbidden. | | |  |  | |  | |  | |  | |  | |  | | | | | |  |
| **III** | More money should be provided for monitoring the execution of animal experiments. | | |  |  | |  | |  | |  | |  | |  | | | | | |  |
|  | Stricter rules should be established for the authorization of animal experiments. | | |  |  | |  | |  | |  | |  | |  | | | | | |  |
|  | The legislature should set a deadline by which science must have managed to completely dispense with animal testing | | |  |  | |  | |  | |  | |  | |  | | | | | |  |
|  | Politics should do more to promote alternative methods. | | |  |  | |  | |  | |  | |  | |  | | | | | |  |
| **Emotional Concerns** | | | | **2.50** | **1.36** | | **-** | | **3.65** | | **1.10** | | **.769** | | **2.30** | | | **1.24** | | **.841** |  |
| **I – III** | Thinking of animals suffering due to medical research makes me sad. | | |  |  | |  | |  | |  | |  | |  | | | | | |  |
| **II+III** | I feel sorry for the animals, which are used in medical tests. | | |  |  | |  | |  | |  | |  | |  | | | | | |  |
| *Note.* Scale from 1 = “do not agree at all” to 5 “agree completely”  *Item was omitted from the index due to a considerable increase in reliability. | | | | | | | | | | | | | | |  | | | | | |  |

Results of Study 1

**S2 Table. Means and Standard Deviations (ANOVA) of Acceptance for Expert Statement and Animal Depiction (Study 1)**

|  | Animal Depiction | | | |
| --- | --- | --- | --- | --- |
|  | Treated | | Untreated | |
| Expert Statement | M | SD | M | SD |
| Necessary | 3.71 | 1.08 | 3.93 | 1.05 |
| Unnecessary | 3.19 | 1.17 | 3.28 | 1.21 |
| *Note.* N = 103; two-way ANOVA; dependent variable: acceptance (scale from 1 to 5).  Expert Statement: F (1,101) = 6.844, p = .010, η² = .065  Animal Depiction: F (1,101) = 0.478, p = .491, η² = .005  Expert Statement x Animal Depiction: F (1, 99) = 0.089, p = .767, η² = .001. | | | | |

**S3 Table. Summary of Univariate Effects on Attitudes Towards Animal Research for Expert Statement and Animal Depiction (Study 1)**

|  | |  | Df | F | η² | p |
| --- | --- | --- | --- | --- | --- | --- |
| Expert Statement | | | | | | |
|  | Moral Justification | | 1 | 4.442 | .044 | .038* |
|  | Necessity | | 1 | 2.963 | .030 | .088 |
|  | Policy Support | | 1 | 3.929 | .039 | .050* |
|  | Emotional Concerns | | 1 | 3.400 | .034 | .068 |
| Animal Depiction | | | | | | |
|  | Moral Justification | | 1 | 0.985 | .010 | .323 |
|  | Necessity | | 1 | 2.248 | .023 | .137 |
|  | Policy Support | | 1 | 2.121 | .021 | .149 |
|  | Emotional Concerns | | 1 | 4.224 | .042 | .043* |
| Expert Statement x Animal Depiction | | | | | | |
|  | Moral Justification | | 1 | 1.307 | .013 | .256 |
|  | Necessity | | 1 | 0.843 | .009 | .361 |
|  | Policy Support | | 1 | 1.746 | .018 | .190 |
|  | Emotional Concerns | | 1 | 0.985 | .010 | .324 |
| Note. N = 101; univariate effects of a two-way MANOVA. *p <.05; **p <.01; ***p <.001  Expert Statement: *F* (4, 94) = 1.343, p = .260, η² = .065  Animal Depiction: *F* (4, 94) = 1.586, p = .184, η² = .063  Expert Statement x Animal Depiction: *F* (4, 94) = 0.476, p = .753, η² = .020. | | | | | | |

**S4 Table. Means and Standard Deviations of Attitudes for Expert Statement and Animal Depiction (Study 1)**

|  | | | Animal Depiction | | | | |
| --- | --- | --- | --- | --- | --- | --- | --- |
|  | | | Treated | | | Untreated | |
|  | Expert Statement | | M | | SD | M | SD |
| DV: Moral Justification | | |  | |  |  |  |
|  | Necessary | | 3.27 | | 0.95 | 3.67 | 0.84 |
|  | Unnecessary | | 3.08 | | 1.15 | 3.05 | 1.04 |
| DV: Necessity | | |  | |  |  |  |
|  | | Necessary | 3.55 | | .63 | 3.93 | .68 |
|  | | Unnecessary | 3.43 | | .85 | 3.52 | .92 |
| DV: Policy Support | | |  | |  |  |  |
|  | Necessary | | 3.40 | | 0.69 | 2.99 | 0.56 |
|  | Unnecessary | | 3.50 | | 0.87 | 3.48 | 0.87 |
| DV: Emotional Concerns | | | |  |  |  |  |
|  | Necessary | | 2.70 | | 1.49 | 1.89 | 0.99 |
|  | Unnecessary | | 2.92 | | 1.55 | 2.64 | 1.55 |
| *Note.* N = 101; scale from 1 to 5 | | | | | | | |

Results of Study 2

**S5 Table. Means and Standard Deviations (ANOVA) of Acceptance for Expert Statement, Animal Depiction and Animal Species (Study 2)**

|  | | | | | | | | |  |
| --- | --- | --- | --- | --- | --- | --- | --- | --- | --- |
|  |  | | Animal Depiction | | | | | |  |
|  |  | | Treated Animal | | | Untreated Animal | | |  |
|  |  | | Rat | Dog | | Rat | | Dog |  |
| Expert Statement | |  | *M (SD)* | | *M (SD)* | | *M (SD)* | *M (SD)* | |
| Necessary | | | 3.09 (1.36) | 3.38 (1.32) | | 3.60 (1.43) | | 3.43 (1.54) |  |
| Unnecessary | | | 3.33 (1.08) | 2.92 (1.36) | | 2.91 (1.47) | | 2.94 (1.18) |  |

| *Note.* N = 434; three-way ANOVA; dependent variable: global acceptance (scale from 1 to 5).  Expert Statement: *F* (1,432) = 7.217, *p* = .008, *η²* = .017  Animal Depiction: *F* (1,432) = 0.099, *p* = .753, *η²* = .000  Animal Species: *F* (1,432) = 0.231, p = .631, *η²* = .001  Expert Statement x Animal Depiction: *F* (1,430) = 3.389, *p* = .066, *η²* = .008.  Expert Statement x Animal Species: *F* (1, 430) = 0.902, *p* = .343, *η²* = .002  Animal Depiction x Animal Species: *F* (1, 430) = 0.001, *p* =.981, *η²* = .000  Expert Statement x Animal Depiction x Animal Species: *F* (1, 426) = 2.881, *p* = .090, *η²* = .007. |
| --- |

**S6 Table. Summary of Univariate Effects of Expert Statement, Animal Depiction and Animal Species on Attitudes Towards Animal Research (Study 2)**

|  | |  | Df | F | η² | P |
| --- | --- | --- | --- | --- | --- | --- |
| Expert Statement | | | | | | |
|  | Moral Justification | | 1 | 3.742 | .009 | .054 |
|  | Necessity | | 1 | 2.530 | .006 | .112 |
|  | Policy Support | | 1 | 3.247 | .008 | .072 |
|  | Emotional Concerns | | 1 | 1.718 | .004 | .191 |
| Animal Depiction | | | | | | |
|  | Moral Justification | | 1 | .047 | .000 | .828 |
|  | Necessity | | 1 | 1.029 | .002 | .311 |
|  | Policy Support | | 1 | .001 | .000 | .972 |
|  | Emotional Concerns | | 1 | .024 | .000 | .877 |
| Animal Species | | |  |  |  |  |
|  | Moral Justification | | 1 | .234 | .001 | .629 |
|  | Necessity | | 1 | 1.169 | .003 | .280 |
|  | Policy Support | | 1 | .463 | .001 | .497 |
|  | Emotional Concerns | | 1 | .823 | .002 | .365 |
| Expert Statement x Animal Depiction | | | | | | |
|  | Moral Justification | | 1 | 7.617 | .018 | .006** |
|  | Necessity | | 1 | 12.310 | .028 | .000*** |
|  | Policy Support | | 1 | 6.719 | .016 | .010** |
|  | Emotional Concerns | | 1 | 3.440 | .008 | .064 |
| Expert Statement x Animal Species | | | | |  |  |
|  | Moral Justification | | 1 | 1.188 | .003 | .276 |
|  | Necessity | | 1 | 1.942 | .005 | .164 |
|  | Policy Support | | 1 | 2.489 | .006 | .115 |
|  | Emotional Concerns | | 1 | .062 | .000 | .803 |
| Animal Depiction x Animal Species | | | | |  |  |
|  | Moral Justification | | 1 | .264 | .001 | .608 |
|  | Necessity | | 1 | .461 | .001 | .497 |
|  | Policy Support | | 1 | 1.516 | .004 | .219 |
|  | Emotional Concerns | | 1 | .346 | .001 | .557 |
| Expert Statement x Animal Depiction x Animal Species | | | | |  |  |
|  | Moral Justification | | 1 | 3.532 | .008 | .061 |
|  | Necessity | | 1 | 3.724 | .009 | .054 |
|  | Policy Support | | 1 | 1.578 | .004 | .210 |
|  | Emotional Concerns | | 1 | .113 | .000 | .737 |
| *Note.* N = 434; three-way ANOVA. *p <.05; **p <.01; ***p <.001  Expert Statement: *F* (4,423) = 1.020, *p* = .396, *η²* = .010  Animal Depiction: *F* (4,423) = 0.805, p = .522, *η²* = .008  Animal Species: *F* (4,423) = 0.605, *p* = .659, *η²* = .006  Expert Statement x Animal Depiction: *F* (4,423) = 3.116, *p* = .014, *η²* = .029  Expert Statement x Animal Species: *F* (4, 423) = 0.890, *p* = .470, *η²* = .008  Animal Depiction x Animal Species: *F* (4, 423) = 0.494, *p* =.740, *η²* = .005  Expert Statement x Animal Depiction x Animal Species: *F* (4, 423) = 1.184, *p* = .317, *η²* = .011 | | | | | | |

**S7 Table. Means and Standard Deviation of Attitudes towards Animal Research for Expert Statement, Animal Depiction and Animal Species (Study 2)**

|  |  | Animal Depiction | | | | | |  |
| --- | --- | --- | --- | --- | --- | --- | --- | --- |
|  |  | Treated Animal | | | Untreated Animal | | |  |
|  |  | Rat | Dog | | Rat | | Dog |  |
| Expert Statement |  | M (SD) | | M (SD) | | M (SD) | M (SD) | |
| DV: Moral Justification | |  |  | |  | |  |  |
| Necessary | | 2.71 (1.06) | 2.92 (1.05) | | 3.16 (1.09) | | 3.08 (1.10) |  |
| Unnecessary | | 3.11 (.96) | 2.70 (1.16) | | 2.60 (1.06) | | 2.68 (1.09) |  |
| DV: Necessity | |  |  | |  | |  |  |
| Necessary | | 2.93 (.89) | 3.06 (.82) | | 3.41 (.83) | | 3.33 (.89) |  |
| Unnecessary | | 3.36 (.86) | 2.94 (.94) | | 2.94 (.87) | | 2.95 (.86) |  |
| DV: Policy Support | |  |  | |  | |  |  |
| Necessary | | 3.88 (.71) | 3.82 (.68) | | 3.70 (.72) | | 3.64 (.85) |  |
| Unnecessary | | 3.63 (.61) | 3.96 (.76) | | 3.98 (.70) | | 3.97 (.67) |  |
| DV: Emotional Concerns | | | | | | | |  |
| Necessary | | 3.63 (1.12) | 3.73 (1.07) | | 3.48 (1.21) | | 3.52 (1.17) |  |
| Unnecessary | | 3.51 (.98) | 3.73 (1.04) | | 3.82 (1.09) | | 3.85 (1.07) |  |

Note. N = 434; three-way ANOVAs, scale from 1 to 5

Results of Study 3

**S8 Table. Means and Standard Deviations (ANOVA) of Acceptance for Expert Statement and Scandalization (Study 3)**

|  | Scandalization | | | |
| --- | --- | --- | --- | --- |
|  | Present | | Absent | |
| Expert Statement | M | SD | M | SD |
| Necessary | 3.38 | 1.31 | 3.50 | 1.19 |
| Unnecessary | 3.11 | 1.29 | 3.24 | 1.10 |
| *Note.* N = 228; two-way ANOVA; dependent variable: acceptance (scale from 1 to 5).  Expert Statement: *F* (1,226) = 2.697, *p* = .102, *η²* = .012  Scandalization: *F* (1,226) = 0.604, *p* = .438, *η²* = .003  Expert Statement x Scandalization: *F* (1,224) = 0.002, *p* = .961, *η²* = .000. | | | | |

**S9 Table. Summary of Univariate Effects of Expert Statement and Scandalization Species on Attitudes Towards Animal Research (Study 3)**

|  | |  | Df | F | η² | p |
| --- | --- | --- | --- | --- | --- | --- |
| Expert Statement | | | | | | |
|  | Moral Justification | | 1 | 0.518 | .002 | .472 |
|  | Necessity | | 1 | 2.296 | .010 | .131 |
|  | Policy Support | | 1 | 2.018 | .009 | .157 |
|  | Emotional Concerns | | 1 | 0.015 | .000 | .903 |
| Scandalisation | | | | | | |
|  | Moral Justification | | 1 | 0.607 | .003 | .437 |
|  | Necessity | | 1 | 1.295 | .006 | .256 |
|  | Policy Support | | 1 | 0.212 | .001 | .645 |
|  | Emotional Concerns | | 1 | 0.296 | .001 | .587 |
| Expertenstatement x Scandalisation | | | | | | |
|  | Moral Justification | | 1 | 1.762 | .008 | .186 |
|  | Necessity | | 1 | 1.300 | .006 | .255 |
|  | Policy Support | | 1 | 0.246 | .001 | .621 |
|  | Emotional Concerns | | 1 | 0.730 | .003 | .394 |
| *N* = 228; two-way MANOVA; *p <.05; **p <.01; ***p <.001  Expert Statement: *F* (4,221) = 1.187, *p* = .318, *η²* = .021  Scandalisation: *F* (4,221) = 0.768, *p* = .547, *η²* = .014  Expert Statement x Scandalisation: *F* (4,221) = 0.499, *p* = .737, *η²* = .009. | | | | | | |

**S10 Table. Means and Standard Deviations of Attitudes for Expert Statement and Scandalisation (Study 3)**

|  | | | Scandalisation | | | | |
| --- | --- | --- | --- | --- | --- | --- | --- |
|  | | | Present | | | Absent | |
|  | Expert Statement | | M | | SD | M | SD |
| DV: Moral Justification | | |  | |  |  |  |
|  | Necessary | | 3.12 | | 1.27 | 3.04 | 1.18 |
|  | Unnecessary | | 2.82 | | 1.09 | 3.13 | .94 |
| DV: Necessity | | |  | |  |  |  |
|  | | Necessary | 3.51 | | 1.15 | 3.51 | 1.05 |
|  | | Unnecessary | 3.14 | | 1.00 | 3.46 | .95 |
| DV: Policy Support | | |  | |  |  |  |
|  | Necessary | | 2.41 | | .90 | 2.40 | .90 |
|  | Unnecessary | | 2.19 | | .84 | 2.30 | .78 |
| DV: Emotional Concerns | | | |  |  |  |  |
|  | Necessary | | 2.43 | | 1.31 | 2.20 | 1.21 |
|  | Unnecessary | | 2.27 | | 1.29 | 2.32 | 1.16 |
| *Note.* N = 228; scale from 1 to 5 | | | | | | | |
